# Towards Green Dentistry: Evaluating the Potential of 4D Printing for Sustainable Orthodontic Aligners with a Reduced Carbon Footprint

**DOI:** 10.3390/polym16243566

**Published:** 2024-12-20

**Authors:** Elena Palmieri, Luca Montaina, Denise Bellisario, Ivano Lucarini, Francesco Maita, Martina Ielmini, Maria Elena Cataldi, Loredana Cerroni, Roberta Condò, Luca Maiolo

**Affiliations:** 1 Institute for Microelectronics and Microsystems, National Research Council, Via del Fosso del Cavaliere, 100, 00133 Rome, Italy; elena.palmieri@artov.imm.cnr.it (E.P.); luca.montaina@artov.imm.cnr.it (L.M.); ivano.lucarini@artov.imm.cnr.it (I.L.); luca.maiolo@cnr.it (L.M.); 2 Department of Industrial Engineering, University of Rome “Tor Vergata”, Via del Politecnico 1, 00133 Rome, Italy; denise.bellisario@unimercatorum.it; 3 Department of Clinical Sciences and Translational Medicine, University of Rome “Tor Vergata”, Via Montpellier, 1, 00133 Rome, Italy; martinaielmini@outlook.com (M.I.); melena.88@hotmail.it (M.E.C.); cerroni@uniroma2.it (L.C.)

**Keywords:** green dentistry, 4D printing, orthodontic aligners, printed polymers, polymers for 4D printing, ClearX, sustainable dentistry

## Abstract

Clear aligners have transformed orthodontic care by providing an aesthetic, removable alternative to traditional braces. However, their significant environmental footprint, contributing to approximately 15,000 tons of plastic waste annually, poses a critical challenge. To address this issue, advancements in 4D printing have introduced “smart” aligners with shape memory properties, enabling reshaping and reducing the number of aligners required per treatment. This study focuses on ClearX aligners, an innovative 4D-printed solution aimed at extending usage duration and minimizing environmental impact. Using a comprehensive suite of tests, including morphological, optical, and mechanical evaluations conducted via scanning electron microscopy, UV-Vis spectroscopy, infrared spectroscopy, and bending and strain assessments, we evaluated the optical and mechanical stability of the ClearX material before and after thermal activation. Our results demonstrate that ClearX aligners retain their structural and functional properties after reshaping. Temporary changes in transparency, observed only under prolonged treatment durations exceeding manufacturer recommendations, are fully reversible within 12 h and do not compromise the aligner’s usability. These findings support the potential of ClearX aligners to effectively combine patient-centered, high-quality orthodontic care with sustainable practices.

## 1. Introduction

Clear aligners have transformed orthodontics, offering advantages over traditional braces, including near invisibility and a removable design for improved oral hygiene. Their origins trace back to 1945, when Kesling proposed a tooth positioning device to guide teeth without bands and wires, which also served as an aligner [1]. In 1971, Ponitz developed a clear plastic appliance using vacuum pressure, emphasizing ease of fabrication and aesthetics [2]. By 1993, Sheridan’s Essix aligners gained popularity [3], paving the way for Invisalign in 1997. This innovation utilized CAD-CAM technology to predict tooth movements and create sequential models for significant repositioning [4,5].

Advancements in 3D printing, introduced in the 1980s, have significantly impacted aligner production by enabling rapid prototyping, cost reduction, and the integration of smart materials with tailored stiffness and biocompatibility [6,7,8,9,10]. However, environmental concerns arise from disposable aligner waste, with an estimated 15,000 tons of plastic generated annually by industry. Approximately 1.2 million aligners are produced daily, many of which end up in landfills or oceans. ClearX has proposed a sustainable alternative using 4D printing to reduce the carbon footprint by half; aligners produce 1.8 kg of CO_2_ compared to the typical 4 kg. This is achieved by exploiting memory-shape properties, allowing aligners to be reshaped and reused during treatment (https://clearxaligners.com/sustainability, accessed on 3 April 2024).

Notably, 4D printing expands on 3D printing by enabling shapeshifting materials that respond to stimuli, introducing scalability, flexibility, and reduced production costs. This approach not only addresses environmental concerns but also enhances aligner functionality, aligning with sustainability goals in orthodontics [11].

So, time represents an additional dimension to model a device and tune its shape.

The study of dynamic shapeshifting in 3D printing (3DP) is leading to advancements in additive manufacturing (AM) thanks to the incorporation of smart materials (SM) into 4D printing (4DP), setting it apart from the rigidity and static nature of traditional 3DP components [12,13,14]. Notably, 4DP introduces transformative possibilities, enabling structures to self-adapt, self-assemble, and self-repair when subjected to external stimuli [15,16].

4DP combines materials, stimuli, and simulations to create programmable systems, with potential applications spanning pharmaceuticals, aerospace, medicine, military, and electronics [17]. Shape-memory materials (SMMs) play a critical role, allowing structures to autonomously reorganize into cohesive forms in response to stimuli such as heat, moisture, or light [18,19,20,21]. This characteristic facilitates the embedding of intricate designs within materials, encouraging bioengineers to explore tissue and organ creation through 4D bioprinting. Biomimetic hydrogels, for example, offer tunable properties for applications in regenerative medicine [22].

The method relies on energy distribution and geometric interactions to achieve dynamic transformations, enabling sustainable and multifunctional material development. As 4DP evolves, its innovative use of self-assembling properties underscores its potential to revolutionize design and manufacturing across diverse fields.

In recent years, 4D materials have been printed using almost any kind of additive manufacturing technique (stereolithography (SLA), selective laser sintering (SLS), fused deposition modeling (FDM), binder jetting 3D printing (BJ), selective laser melting (SLM), direct ink writing (DIW), etc.) by using materials responsive to moisture like hydrogel or responsive to temperature (thermo-responsive materials) or to light (photo-responsive) or to electric or magnetic energy (electro- or magneto responsive). So, electricity, heat, light, or water can be stimuli for 4DP. This technique makes use of the capabilities found in small structures. When appropriately planned, the desired macro-structure deformation becomes visible [23,24]. With these methodologies, scholars have recently demonstrated prototypes in the electronics and aerospace sectors with the fabrication of self-foldable solar cells [25], in soft robotics [26], in medical science with the development of smart stents [27] and 4D bioprinting for the implementation of smart drug delivery systems or tissue regeneration exploiting the self-repair capability of these materials [28,29]. Notably, 4DP can be used in dentistry for removable prosthetics. Materials that resemble the hard and soft natural tissues found in dentistry can be produced using this technology. Additionally, 4D-printed materials could be modified to accommodate oral cavity forces. Due to their self-folding nature, the pre-printed 4D materials provide optimal dynamic properties as well as reliable fitting and retention capabilities. It is possible to incorporate certain structures into the denture foundation that have temperature and flexibility characteristics like those of periodontal ligaments or excessive mucus [30,31,32]. In dentistry, and particularly in orthodontics, 4D printing has introduced innovative concepts for creating devices that combine optimal functionality with sustainability, aligning with the principles of green dentistry.

Green dentistry emphasizes reducing the environmental impact of dental practices using eco-friendly materials and sustainable methods. Incorporating non-toxic, biocompatible materials and minimizing exposure to harmful chemicals enhance patient safety and promote a cleaner clinical environment. This approach encourages water and energy conservation in oral healthcare routines while preparing dental practices to comply with evolving environmental regulations, thus avoiding potential penalties and reducing operational costs.

In this context, ClearX aligners represent a notable innovation, offering orthodontic treatments with a consistent reduction in the carbon footprint of traditional clear aligners. These aligners leverage 4D printing technology, allowing reshaping through thermal activation—patients can reuse the same device after reshaping it in boiling water before transitioning to the next stage of treatment.

The purpose of this work is to verify that the aligners introduced by ClearX do not lose their mechanical and optical properties without undergoing chemical degradation after being reshaped, thus ensuring the correct operation of the orthodontic device for the following two weeks. In this respect, extensive work has been performed to evaluate morphological changes on the sample surface and on its transparency, by implementing scanning electron microscopy, UV-Vis spectroscopy, and infrared spectroscopy. In addition, the mechanical stability of these polymers has been studied to examine possible differences prior to and following the aligner reshaping.

## 2. Materials and Methods

ClearX samples coming from different disks provided directly by the manufacturer were cut with a cutter into strips of 5.0 × 0.5 cm^2^ and stored in a controlled environment with fixed relative humidity (40%) and temperature (21 °C). The manufacturer recommends a minimum treatment time of 10 min; however, we extended the treatment duration to account for possible carelessness by the end user. For each experiment, we used a set of 5 samples treated under the same conditions (untreated, 3, 10, 30, 60, and 120 min), reporting the average and standard deviation of each analysis.

UV-Vis spectroscopy was performed to assess the transparency of the aligners before and after the treatment. UV-Vis spectroscopy was conducted using a Perkin Elmer (Waltham, MA, USA) Lambda 35 UV/Vis spectrometer. Spectra were recorded from 1100 to 200 nm with a resolution of 4 nm.

FTIR-ATR characterization was carried out using a Thermo Fisher Scientific (Waltham, MA, USA) Nicolet Summit Pro spectrometer in absorbance mode. Measurements were performed in attenuated total reflectance mode (ATR) with a diamond crystal single reflection accessory. Spectra were collected from 4000 to 400 cm^−1^ with a resolution of 2 cm^−1^. Thirty-two scans for each sample were taken.

Mechanical characterization was performed using a Newport (Irvine, CA, USA) SP300 motorized linear stepping motor. Material samples, cut into 4 × 0.5 cm^2^ pieces, were mounted on the linear trail and subjected to bending cycles with an end-to-end distance of 1.5 cm. One end of the sample was fixed, while the other was moved back and forth cyclically at a fixed speed of 2 mm s^−1^. The motor was controlled by custom LabView software, which managed the advance and retraction of the movable holder.

The Shore A hardness, which measures the hardness of flexible mold rubbers, was measured by means of a digital durometer model Candeonxnh52kavou following the ISO 868:2003 standard [33].

The morphology of the aligner sample subjected to bending tests was monitored every 100 bending cycles to observe any changes and was characterized via scanning electron microscopy using an FE-SEM (Sigma 300 Carl Zeiss, Oberkochen, Germany) with an intensity of 10 kV, in HV mode, using secondary electrons, and via optical microscopy with an optical Olimpus (Tokyo, Japan) mx50 microscope.

The thickness of the samples was measured using a digital micrometer by Mitutoyo (Sakado, Japan), able to measure thicknesses from 1 µm to 25 mm with an accuracy of 1 µm.

## 3. Results

The invisible orthodontic aligners were immersed in boiling water (100 °C) for different time intervals (3, 10, 30, 60, and 120 min) and compared with untreated samples to evaluate potential changes in morphology, chemical composition, transparency, and material hardness.

**Figure 1:**
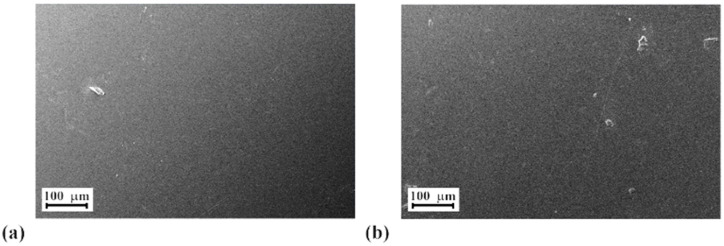
Secondary electrons SEM images of (**a**) untreated (**b**) sample treated for 30 min.

Morphological investigation carried out via SEM microscopy (Figure 1a,b) revealed no discernible differences between untreated (a) and treated (b) samples.

**Figure 2:**
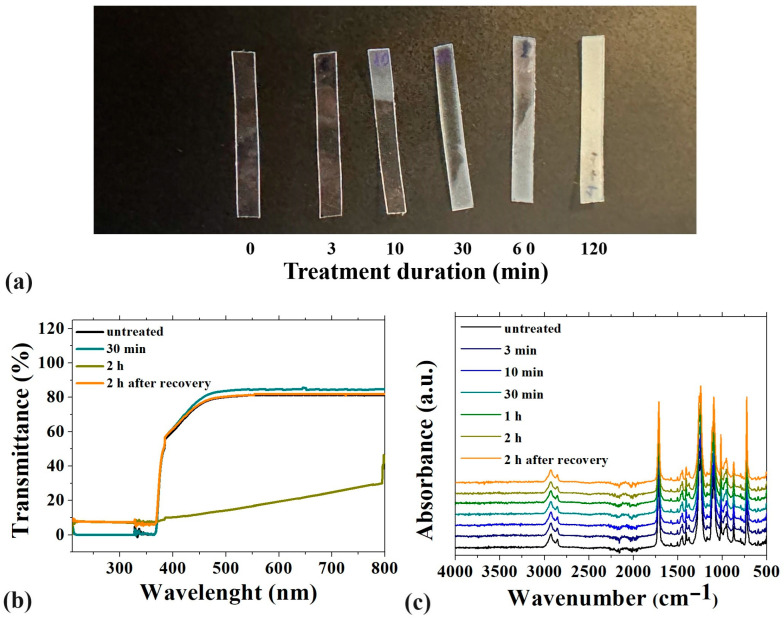
(**a**) Picture of untreated and treated samples, (**b**) UV-VS spectra of untreated and treated aligners, (**c**) ATR-FTIR spectra of the orthodontic aligners.

No significant changes were observed in the UV-Vis spectra for treatments up to 60 min, although initial signs of opacification appeared at the sample’s margins after 30 min (Figure 2a). After 120 min, a noticeable loss of transparency was evident (Figure 2a), and it was confirmed by the UV-Vis data (Figure 2b). However, the material fully regained its original transparency after resting overnight (around 12 h) at room temperature (Figure 2b, yellow line).

To best interpret the ATR-FTIR spectra, one should consider that, as demonstrated by previous research [34], polymers commonly used for clear aligners are thermoplastic polyurethane (TPU), polyethylene terephthalate glycol-modified (PETG), polyethylene terephthalate (PET), polypropylene, polycarbonate (PC), poly(cyclohexylenedimethylene) terephthalate (PCT), ethylene vinyl acetate, and others.

As declared by the manufacturer, ClearX belongs to the chemical family of copolyester-polyurethane materials. From the ATR-FTIR spectra reported in Figure 2c, a typical peak of polymers of the PU family at 3300 cm^−1^ associated with the NH bond stretching is not observed. This peak is usually broad due to hydrogen bonding in the urethane group. The peak at 1500 cm^−1^ related to the amide-II band is small. The extent of hydrogen bonding or phase segregation in the material may contribute to variations from the “standard” urethane spectra. The precise positions and intensities of the FTIR peaks can vary based on the specific formulation of the polyurethane, influenced by the types of diisocyanates and polyols used. This variability could also arise because the aligner material is a copolymer, introducing further complexity to its molecular structure and interactions. The 1700 cm^−1^ and 1250 cm^−1^ peaks can be associated with PETG, TPU, and PCT-based thermal plastics, respectively, the C=O stretching at around 1700 cm^−1^, and the bending vibration of -CH_2_ groups at around 1340 and 1180 cm^−1^. Other typical peaks associated with polymers from the polyester and polyurethane families include the CH_2_ stretching band around 2900 cm^−1^, and the vibration of an aromatic ring at around 1590 cm^−1^ and 750–699 cm^−1^ [34].

Post-treatment, no changes were observed in any region of the spectra, including the 3400 cm^−1^ region associated with OH stretching vibrations of absorbed water molecules. Therefore, ATR-FTIR spectra suggest that no chemical reaction or water uptake occurred, even after 120 min in boiling water. The temporary loss in transparency is likely due to the transient swelling of the polymer structure, which reverts to its original state within 24 h.

**Figure 3:**
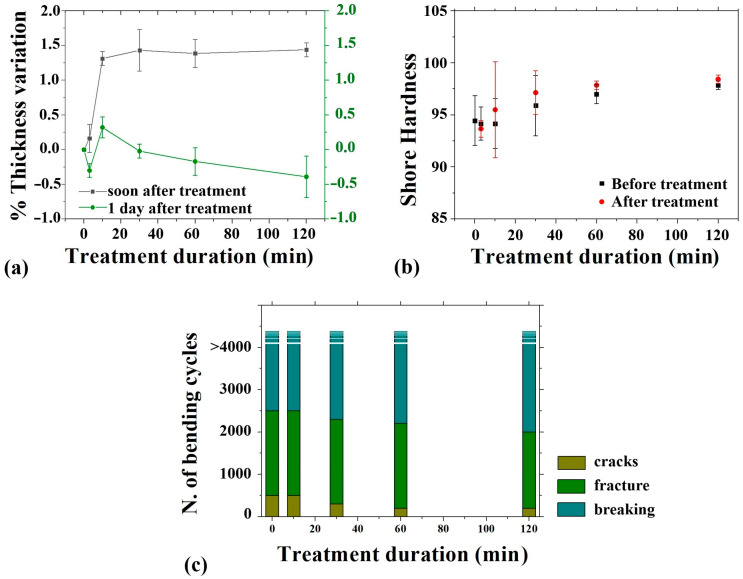
(**a**) Relative thickness variation of the materials, (**b**) Hardness of the material, (**c**) Bending test results.

The thickness of the aligner material was measured, and the percentage variation is shown in Figure 3a. A slight increase in thickness was observed after the treatment, and values returned to the initial values after recovery at room temperature overnight.

**Figure 4:**
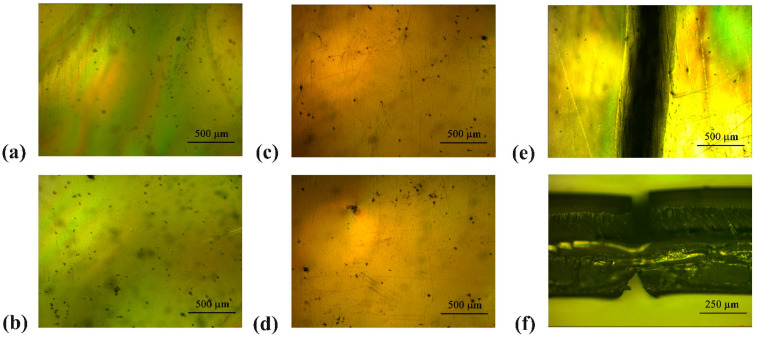
Optical microscope images of the samples (**a**) untreated; (**b**) 120 min-treated in hot water; (**c**) untreated, after 500 bending cycles; (**d**) 120 min-treated in hot water after 200 bending cycles; fracture point of the untreated sample (**e**) above view and (**f**) side view.

Regarding the material’s hardness, no significant changes were observed (Figure 3b), as the minor variations fell within the margin of error and were deemed statistically insignificant. The slightly larger error margins noted in the samples treated for 10 and 30 min could be attributed to ongoing structural adjustments during the initial phase of treatment. After this period, the process appears to stabilize, resulting in more uniform measurements. Nonetheless, as these differences remain minimal and within acceptable error ranges, definitive conclusions cannot be established. The mechanical performance of the aligners was assessed through bending tests, with the results shown in Figure 3c. The morphology of the aligners during bending tests was monitored and SEM images are reported in Figure 4a–f. All aligner samples, including untreated ones and those treated in hot water for 10, 30, 60, and 120 min, underwent 4000 bending cycles without breaking into two separate parts. However, scattered cracks and a fracture at the point of maximum bending were observed at various stages, depending on the sample’s treatment condition. For the untreated samples, the fracture occurred after 2500 cycles, while cracks started forming after 500 cycles. These observations are detailed in Figure 3c and illustrated further in Figure 4a,c,e,f. Specifically, Figure 4a shows the intact flat surface of the untreated aligner. Figure 4c depicts the onset of cracks, while Figure 4e,f capture the main fracture that emerged after 2500 cycles. For samples treated in hot water for 10 and 30 min, fractures were observed after approximately 2500 and 2300 cycles, respectively. Cracks began to appear at 300 cycles for the 10-min-treated samples and at 200 cycles for the 30-min-treated ones (Figure 3c). The 60-min-treated samples exhibited cracks after 200 cycles, with fractures developing after 2200 cycles. Similarly, the samples treated for 120 min began forming cracks at 200 cycles (as shown in Figure 4d) and fractured after 2200 cycles.

## 4. Discussion

Smart memory polymers could transform clear aligner fabrication by enabling devices that support two treatment phases, potentially halving plastic usage while retaining clinical effectiveness. Clear aligners, preferred for their comfort and aesthetics, have seen growing demand, especially among adults. Made from materials like PET-G and TPU, they are valued for their affordability, durability, and ease of production, with low energy requirements and excellent chemical stability [20]. Plastic, though highly adaptable, is now recognized for its environmental and health impacts as a single-use material. Reducing plastic use requires minimizing production and enhancing recycling. In orthodontics, alternatives to plastic for aligner manufacturing are unlikely, but shape memory polymers could lower plastic consumption by reducing the number of aligners needed per treatment, contributing to a ‘greener’ orthodontic correction [21].

The use of shape memory polymers in orthodontic aligners aligns with the Global Oral Health Action Plan (2023–2030), which prioritizes sustainability in oral healthcare. By reducing the number of aligners needed, this innovation helps minimize plastic waste, supporting the plan’s objective of decreasing reliance on single-use plastics and promoting eco-friendly healthcare practices.

This in vitro study examines the chemical-physical stability of orthodontic aligners made from thermo-responsive smart memory polymers. The material’s characteristics were analyzed before and after treatment in boiling water over various time intervals. Aligners typically degrade at 300 °C, while the glass transition temperature is around 80 °C and the melting temperature exceeds 150 °C [35]. At 100 °C, only mild changes in material structure are expected. Therefore, the study aims to assess whether permanent changes occur in the polymer, impacting its clinical function, with the null hypothesis assuming no transformation after heat treatment.

The study of ClearX material confirmed that the aligners exhibited no significant surface or structural changes after undergoing the heat treatment process in boiling water, which activates the shape memory effect. The chemical-physical stability was evaluated, extending beyond the manufacturer’s recommended time interval, with testing conducted for up to two hours.

Physical stability was assessed through hardness measurements, taken before and after treatment for durations of 3, 10, 30, 60, and 120 min. The results showed that the treatment did not alter the material’s ability to resist permanent deformation, confirming that the aligners maintained their plastic deformability throughout the process.

To evaluate potential changes in transparency and chemical properties, UV-Vis and FTIR spectroscopy were used on the samples. Transparency is a key aesthetic feature for orthodontic aligners, as it is important for adult patients who prefer less noticeable treatments compared to metal braces. The analysis revealed no significant change in the optical properties up to 60 min of treatment, though minor signs of opacity were visually observed. After 120 min, a more noticeable loss of transparency was recorded, but the material regained its original clarity after resting overnight at room temperature. This temporary opacity was likely caused by swelling of the polymer structure, which returned to its original state within 24 h, as confirmed by a slight increase in thickness that normalized by the following day. When a polymer is exposed to a solvent and subjected to heat, it may undergo a swelling process. During this phenomenon, the polymer’s structure relaxes, creating voids within the polymeric network that allow small molecules from the solvent to penetrate. Although this process does not involve chemical modification of the polymer’s molecular structure—making it undetectable through FTIR analysis—it can induce changes in the material’s physical-chemical properties, including a slight increase in size. Typically, this swelling is a reversible process; removing the solvent enables the polymer to revert to its original conformation, restoring its initial dimensions and characteristics [36,37].

Overall, the material exhibited excellent stability in both its physical and optical properties, with any temporary changes fully reversing after a short period.

In this context, infrared spectroscopy with Fourier transform has made it possible to assess whether chemical changes occur in the context of the polymer material following the treatment, that could potentially lead to an increase in the rate of degradation, affecting the longevity and performance of aligners in terms of clinical efficacy. Analysis of the obtained FTIR-ATR shows that no chemical reaction or chemical water absorption occurred, even after the sample was immersed in water at 100 °C for 120 min.

Finally, by means of SEM investigations, it was possible to observe the surface of samples subjected to heat treatment at 100 °C and to compare it with the surface of untreated samples, for the purpose of analyzing whether the above-mentioned process may have some influence on the surface morphology of the material. The survey did not reveal any difference in surface morphology between treated and untreated samples. It has thus been shown that the material subjected to heat treatment is free from any kind of impurity on its surface. Even for the mechanical bending tests, the samples related to the untreated devices and the samples treated for 10 and 30 min do not show any significant difference in terms of cracks and fractures, while for longer treatment a slight reduction in mechanical performance is visible, but these conditions are far from the clinical protocol proposed by the manufacturer and the samples have been subjected to a mechanical stress that can unlikely occur during the treatment.

## 5. Conclusions

In conclusion, we demonstrated that the 4D material used in ClearX maintains its mechanical and optical properties after its shapeshifting. The initial hypothesis is confirmed, as the material shows no significant surface or structural changes after being treated in boiling water for two hours—far exceeding the manufacturer’s recommended treatment time of 10 to 30 min outlined in the clinical protocol. Temporary changes in transparency induced by prolonged treatments do not last more than 12 h. All these findings have been observed through deep optical and mechanical characterization based on optical and scanning electron microscopy, FTIR, UV-Vis spectroscopy, hardness tests, and mechanical bending tests. These results are very important since smart memory polymers offer significant promise for producing clear orthodontic aligners that enhance sustainability and promote patient awareness of materials used in clinical treatments. This approach empowers patients to select eco-friendly solutions aligned with green dentistry guidelines, integrating high-quality healthcare with environmentally conscious choices.

## Data Availability

The original contributions presented in this study are included in the article. Further inquiries can be directed to the corresponding authors.

## References

[1] Kesling H.D. (1945). The philosophy of the tooth positioning appliance. Am. J. Orthod. Oral Surg..

[2] Ponitz P.V. (1957). The relative importance of appliances in orthodontic therapy. J. Am. Dent. Assoc..

[3] Sheridan J., LeDoux W., McMinn R. (1993). Essix retainers: Fabrication and supervision for permanent retention. J. Clin. Orthod..

[4] Lagravère M.O., Flores-Mir C. (2005). The treatment effects of Invisalign orthodontic aligners. J. Am. Dent. Assoc..

[5] AlMogbel A. (2023). Clear Aligner Therapy: Up to date review article. J. Orthod. Sci..

[6] Liaw C.-Y., Guvendiren M. (2017). Current and emerging applications of 3D printing in medicine. Biofabrication.

[7] Yi H.-G., Lee H., Cho D.-W. (2017). 3D Printing of Organs-On-Chips. Bioengineering.

[8] Dawood A., Marti B.M., Sauret-Jackson V., Darwood A. (2015). 3D printing in dentistry. Br. Dent. J..

[9] Oberoi G., Nitsch S., Edelmayer M., Janjić K., Müller A.S., Agis H. (2018). 3D Printing—Encompassing the Facets of Dentistry. Front. Bioeng. Biotechnol..

[10] Condo R., Pazzini L., Cerroni L., Pasquantonio G., Lagana G., Pecora A., Mussi V., Rinaldi A., Mecheri B., Licoccia S. (2018). Mechanical properties of ‘two generations’ of teeth aligners: Change analysis during oral permanence. Dent. Mater. J..

[11] Tibbits S. (2014). 4D Printing: Multi-Material Shape Change. Archit. Des..

[12] Kumar S.B., Jeevamalar J., Ramu P., Suresh G., Senthilnathan K. (2021). Evaluation in 4D printing—A review. Mater. Today Proc..

[13] Yousuf M.H., Abuzaid W., Alkhader M. (2020). 4D printed auxetic structures with tunable mechanical properties. Addit. Manuf..

[14] McLellan K., Sun Y.-C., Naguib H.E. (2022). A review of 4D printing: Materials, structures, and designs towards the printing of biomedical wearable devices. Bioprinting.

[15] Goo B., Hong C.-H., Park K. (2020). 4D printing using anisotropic thermal deformation of 3D-printed thermoplastic parts. Mater. Des..

[16] Ahmed A., Arya S., Gupta V., Furukawa H., Khosla A. (2021). 4D printing: Fundamentals, materials, applications and challenges. Polymer.

[17] Momeni F., Hassani N.S.M.M., Liu X., Ni J. (2017). A review of 4D printing. Mater. Des..

[18] Wu D., Leng Y.-M., Fan C.-J., Xu Z.Y., Li L., Shi L.Y., Yang K.-K., Wang Y.-Z. (2022). 4D Printing of a Fully Biobased Shape Memory Copolyester via a UV-Assisted FDM Strategy. ACS Sustain. Chem. Eng..

[19] Ntouanoglou K., Stavropoulos P., Mourtzis D. (2018). 4D Printing Prospects for the Aerospace Industry: A critical review. Procedia Manuf..

[20] Baumgartner M., Hartmann F., Drack M., Preninger D., Wirthl D., Gerstmayr R., Lehner L., Mao G., Pruckner R., Demchyshyn S. (2020). Resilient yet entirely degradable gelatin-based biogels for soft robots and electronics. Nat. Mater..

[21] Velu R., Tulasi R., Ramachandran M.K., Deshmukh K., Pasha S.K.K., Sadasivuni K.K. (2023). Environmental Impact, Challenges for Industrial Applications and Future Perspectives of Additive Manufacturing. Nanotechnology-Based Additive Manufacturing.

[22] Gladman A.S., Matsumoto E.A., Nuzzo R.G., Mahadevan L., Lewis J.A. (2016). Biomimetic 4D printing. Nat. Mater..

[23] Liu G., He Y., Liu P., Chen Z., Chen X., Wan L., Li Y., Lu J. (2020). Development of Bioimplants with 2D, 3D, and 4D Additive Manufacturing Materials. Engineering.

[24] Manikandan N., Rajesh P.K., Harish V. (2021). An analysis of the methods and materials for 4-dimensional printing. Mater. Today Proc..

[25] Momeni F., Ni J. (2018). Nature-inspired smart solar concentrators by 4D printing. Renew. Energy.

[26] Hann S.Y., Cui H., Nowicki M., Zhang L.G. (2020). 4D printing soft robotics for biomedical applications. Addit. Manuf..

[27] Zarek M., Mansour N., Shapira S., Cohn D. (2017). 4D Printing of Shape Memory-Based Personalized Endoluminal Medical Devices. Macromol. Rapid Commun..

[28] Kang H.-W., Lee S.J., Ko I.K., Kengla C., Yoo J.J., Atala A. (2016). A 3D bioprinting system to produce human-scale tissue constructs with structural integrity. Nat. Biotechnol..

[29] Cui H., Liu C., Eswothy T., Huang Y., Yu Z.X., Zhou X., San H., Lee S.-J., Hann S.Y., Boehm M. (2020). 4D physiologically adaptable cardiac patch: A 4-month in vivo study for the treatment of myocardial infarction. Sci. Adv..

[30] Piedra-Cascón W., Krishnamurthy V.R., Att W., Revilla-León M. (2021). 3D printing parameters, supporting structures, slicing, and post-processing procedures of vat-polymerization additive manufacturing technologies: A narrative review. J. Dent..

[31] Li Y., Zhang F., Liu Y., Leng J. (2020). 4D printed shape memory polymers and their structures for biomedical applications. Sci. China Technol. Sci..

[32] Sharma D., Mathur V.P., Satapathy B.K. (2021). Biodegradable and Biocompatible 3D Constructs for Dental Applications: Manufacturing Options and Perspectives. Ann. Biomed. Eng..

[33] (2003). Plastics and Ebonite—Determination of Indentation Hardness by Means of a Durometer (Shore Hardness).

[34] Rusu E., Drobota M., Barboiu V. (2008). Structural investigations of amines treated polyester thin films by FTIR-ATR spectroscopy. J. Optoelectron. Adv. Mater..

[35] Kwok M., Porto B., Mohebi S., Zhu L., Hans M. (2022). Physical and chemical properties of five different clear thermoplastic materials. J. Appl. Polym. Sci..

[36] Zheng W., Liu C., Wei X., Sun W., Zhao L. (2023). Molecular-level swelling behaviors of poly (ethylene terephthalate) glycolysis using ionic liquids as catalyst. Chem. Eng. Sci..

[37] Ahad N.A. (2020). A Recent blend of thermoplastic polyurethane (TPU). IOP Conf. Ser. Mater. Sci. Eng..

